# Transarterial chemoembolization combined with recombinant human adenovirus type 5 H101 prolongs overall survival of patients with intermediate to advanced hepatocellular carcinoma: a prognostic nomogram study

**DOI:** 10.1186/s40880-017-0227-2

**Published:** 2017-07-20

**Authors:** Chao-Bin He, Xiang-Ming Lao, Xiao-Jun Lin

**Affiliations:** Department of Hepatobiliary Oncology, State Key Laboratory of Oncology in South China, Collaborative Innovation Center for Cancer Medicine, Sun Yat-sen University Cancer Center, Guangzhou, 510060 Guangdong P. R. China

**Keywords:** Transarterial chemoembolization, Recombinant human adenovirus type 5, Hepatocellular carcinoma, Prognosis, Nomogram

## Abstract

**Background:**

Patients with intermediate to advanced hepatocellular carcinoma (HCC) are most commonly treated with transarterial chemoembolization (TACE). Previous studies showed that TACE combined with recombinant human adenovirus type 5 (H101) may provide a clinical survival benefit. In the present study, we aimed to determine the survival benefit of TACE with or without H101 for patients with intermediate to advanced HCC and to develop an effective nomogram for predicting individual survival outcomes of these patients.

**Methods:**

We retrospectively collected data from 590 patients with intermediate to advanced HCC who were treated at Sun Yat-sen University Cancer Center between January 2007 and July 2015. After propensity score matching, 238 patients who received TACE with H101 (TACE with H101 group) and 238 patients who received TACE without H101 (TACE group) were analyzed. Overall survival (OS) was evaluated using the Kaplan–Meier method; the nomogram was developed based on Cox regression analysis. Discrimination and calibration were measured using the concordance index (c-index) and calibration plots.

**Results:**

Clinical and radiologic features were similar between the two groups. OS rates were significantly lower in the TACE group than in the TACE with H101 group (1-year OS rate, 53.8% vs. 61.3%; 2-year OS rate, 33.4% vs. 44.2%; 3-year OS rate, 22.4% vs. 40.5%; all *P* < 0.05). Multivariate Cox regression analysis for the entire cohort showed that alpha-fetoprotein level, alkaline phosphatase level, tumor size, metastasis, vascular invasion, and TACE with or without H101 were independent factors for OS, all of which were included in the nomogram. Calibration curves showed good agreement between nomogram-predicted survival and observed survival. The c-index of the nomogram for predicting OS was 0.716 (95% confidence interval 0.686–0.746).

**Conclusions:**

TACE plus H101 extends the survival of patients with intermediate to advanced HCC. Our proposed nomogram provides individual survival prediction and stratification for patients with intermediate to advanced HCC who receive TACE with or without H101.

## Background

Liver cancer is prevalent in East and Southeast Asia and in North and West Africa [1]. In China, it accounts for 13.79% of all cancer cases and its mortality ranks the third among all cancers [2]. Worldwide, hepatocellular carcinoma (HCC) is the major type of liver cancer, which is the fifth most common cancer and the third leading cause of cancer-related death [3, 4]. Hepatectomy, liver transplantation, and radiofrequency ablation are recommended as curative approaches for patients with early-stage HCC [5–7]. Other patients who present with portal vein tumor invasion or multiple tumors, which are categorized as Barcelona Clinic Liver Cancer stage B and C, are diagnosed with intermediate to advanced HCC. Transarterial chemoembolization (TACE), which allows chemotherapeutics to focus on the tumor while blocking tumor-feeding arteries, is most commonly used for patients with intermediate to advanced HCC [8–10]. Because of diminished liver function, enlarged tumors, and portal vein involvement, TACE becomes less effective when administered for longer than 6 months and is associated with unfavorable prognosis [11]. Furthermore, because of liver ischemia and hypoxia which can up-regulate the expression of vascular endothelial growth factor and enhance angiogenesis, TACE can actually promote tumor growth [12, 13]. The prognosis of patients with intermediate to advanced HCC who are treated with TACE is unfavorable, with a median overall survival (OS) of only 6.7 months [14]. This indicates that new treatment strategies for intermediate to advanced HCC are necessary.

As shown in past studies, genetic abnormalities were presented in HCC cells, including the activation of oncogenes [15] and the inactivation of tumor suppressor genes [16]. Mutations of the tumor protein P53 (*TP53*) gene frequently happen during tumor development. The P53 protein has many functions, including arrest of the G_1_ phase of cell cycle via the cyclin-dependent kinase inhibitor P21^Cip1^ and induction of apoptosis via inducing the expression of apoptosis-related genes, such as Bcl-2-associated X protein (*BAX*). Consequently, P53 plays an important role in tumor apoptosis and necrosis [17]. Mutations or deletions of wild-type *TP*53 exist in nearly 50% of all tumor types [18] and in 30%–50% of HCCs [19] and indicate an unfavorable prognosis for patients with HCC. The administration of recombinant adenovirus p53 (rAd-p53) can improve the prognosis of cancer patients [20–23]. Furthermore, the combination of TACE and rAd-p53 may be superior to TACE alone in delaying the progression of HCC and extending the survival of patients with HCC [24–26]. H101, recombinant human adenovirus type 5 (rAd5), is generated by deleting the entire *E1B* gene and partial *E3* gene in the viral genome [20]. H101 selectively replicates in cancer cells but cannot replicate in normal cells or lyse normal cells where P53 is active without E1B to inactivate P53 [27]. The segment of *E3* gene in H101, which encodes the death protein, is deleted to potentially improve the safety of this virus product [20]. H101 infects tumors and finally kills tumor cells through viral oncolysis [27, 28]. Some studies showed that H101 replicates in many tumor cell lines regardless of the P53 status [17, 29–31]. The activated host immune system and enhanced cell-medicated immune responses induced by H101 injection may contribute to tumor regression, thereby enhancing the antitumor effect of TACE treatment [32].

Some studies showed that H101 is safe and efficacious via transarterial infusion when combined with TACE in patients with intermediate to advanced HCC [33, 34]. However, the heterogeneity of the study populations led to variations in OS estimation. Moreover, it is difficult to stratify the prognosis of patients according to their risk level. Therefore, it is essential to develop a predictive system that incorporates parameters associated with the prognosis of patients with intermediate to advanced HCC who received TACE with H101.

Nomograms are statistical models developed specifically for individual and highly accurate estimation of prognosis [35]. Furthermore, nomograms can improve the prognosis-predicting abilities of staging systems from the population level to the individual level; this indicates that researchers can use nomograms to calculate the approximate survival probability of individual patients at the time of diagnosis. In the present study, we aimed to develop a prognostic nomogram for patients with intermediate to advanced HCC after initial treatment with TACE with or without H101.

## Patients and methods

### Patients

Patients who were newly diagnosed with intermediate to advanced HCC and those who underwent TACE with or without H101 as initial therapy at the Department of Hepatobiliary Oncology of Sun Yat-sen University Cancer Center (Guangzhou, China) between January 2007 and July 2015 were included. Inclusion criteria were as follows: (1) three courses of TACE with or without H101 as the only treatment and (2) pre-TACE liver function of Child–Pugh A or Child–Pugh B. Exclusion criteria were as follows: (1) other treatments, including hepatic resection, radiofrequency treatment, and liver transplantation for HCC before or after TACE; (2) inadequate renal function (serum creatinine level and blood urea nitrogen level higher than the upper limits of normal); (3) severe coagulopathy (prothrombin activity <40% or platelet count <40,000/mm^3^); (4) Child–Pugh C liver function or evidence of hepatocellular decompensation, including refractory ascites, esophageal or gastric variceal bleeding, and hepatic encephalopathy; (5) obstructive jaundice; (6) other concurrent primary tumors; or (7) lost to follow-up. All patients were followed up for at least 1 year after treatment. This study was approved by the Institutional Review Board of Sun Yat-sen University Cancer Center. All procedures performed in the present study involving human participants were in accordance with the ethical standards of institutional and/or national research committees and the 1964 Helsinki Declaration and its later amendments or similar ethical standards.

### Clinical data collection

All clinical and radiologic data for diagnosis were retrieved from medical records archived at Sun Yat-sen University Cancer Center. Hematologic and imaging examinations, including alpha-fetoprotein (AFP) test, liver ultrasonography, magnetic resonance imaging (MRI), contrast-enhanced dynamic computed tomography (CT), and hepatic arterial angiography, were used in the clinical diagnosis of HCC. Diagnoses were based on the findings of typical radiologic features in at least two imaging examinations, histological confirmation by needle biopsy, or a single positive imaging diagnosis combined with elevated serum AFP level [36]. Vascular invasion was defined as the presence of thrombus adjacent to the tumor in the portal system or hepatic vein system with a vague boundary, confirmed by at least two imaging modalities [37].

The following clinical and radiologic data were collected and analyzed: age, sex, white blood cell count, platelet count, neutrophil cell count, lymphocyte count, serum levels of AFP, alanine transaminase (ALT), aspartate aminotransferase (AST), total bilirubin (TBIL), indirect bilirubin (IBIL), alkaline phosphatase (ALP), albumin (ALB), C-reactive protein (CRP), and hepatitis B surface antigen (HBsAg), neutrophil-to-lymphocyte ratio (NLR), hepatitis B virus (HBV)-DNA load, splenomegaly, tumor number, vascular invasion, antiviral therapy, and tumor size (which refers to tumor diameter of the single tumor or the largest tumor when patients presented with multiple tumors).

### Treatment procedure

For each patient, a uniform treatment protocol was performed. All selected patients received three courses of TACE with or without H101. The Seldinger technique for TACE was used as previously described [38]. Conventional chemoembolization was performed by administering 300 mg carboplatin, 50 mg epirubicin, and 6 mg mitomycin. Depending on tumor location, tumor size, and number of tumors, the dose of lipiodol delivered ranged from 5 mL to 30 mL. Chemotherapeutic agents suspended in lipiodol were injected as selectively as possible into the hepatic segmental artery where the target tumor was located.

For patients treated with TACE with H101, H101 was administered before the injection of chemotherapeutic agents via the catheter into the hepatic artery supplying the tumor(s). A total of 1.0 × 10^12^ virus particles in 10 mL of 0.9% sodium chloride solution were administered. Aseptically purified particles of virus H101 were produced for human clinical use by Shanghai Sunway Biotech (Shanghai, China). The titer, sterility, and general safety were tested by the National Institute for the Control of Pharmaceutical and Biological Products (Beijing, China) [39].

### Follow-up

After the first course of TACE, patients were followed up at least every 2 months during the first year and every 3 months thereafter. AFP test, liver ultrasonography, CT, and MRI were selectively performed as needed. OS was defined as the duration (in months) from the date of the first course of TACE until death or the last follow-up. The last follow-up was completed on August 31, 2016.

### Statistical analysis

To minimize potential selection bias, patients treated with TACE alone were selected to match those treated with TACE with H101 using the propensity score matching. Patients were matched according to age, sex, tumor size, tumor number, metastasis, vascular invasion, and splenomegaly. The optimal cutoff value for NLR was determined using time-dependent receiver operating curve (ROC) analysis, which was performed using the package “survivalROC” in R version 3.2.5. The nearest 1:1 neighbor method without replacement was applied to match patients in the TACE group to patients in the TACE with H101 group. After matching, the Chi square test was used to compare the two groups for homogeneity. Propensity score matching analysis was performed using the “MatchIt” package in R version 3.2.5 (R Foundation for Statistical Computing, Vienna, Austria).

SPSS version 22 software (SPSS Inc., Chicago, IL, USA) was used to analyze the data. Continuous variables are expressed as means with standard deviations or medians with ranges and were compared between the TACE with H101 group and the TACE group using the two-tailed unpaired *t* test. The chi square test and Fisher’s exact test were used to compare categorical variables, which are presented as the numbers of patients and percentages.

The patients who were alive at last follow-up, died of non-cancer related causes, or lost to follow-up were censored. OS curves were analyzed using the Kaplan–Meier method, and differences between groups were identified using the log-rank test. Analyses for survival curves were performed using MedCalc software version 11.4.2.0 (MedCalc, Ostend, Belgium). Univariate analysis was performed to assess significance of clinical and radiologic characteristics. Multivariate analysis was performed using the Cox regression model for variables found to be significant in univariate analysis, and the corresponding 95% confidence intervals (CIs) were calculated. Two-tailed *P* values less than 0.05 were considered statistically significant.

A nomogram was developed based on the independent risk factors identified in the multivariate analysis, using the package *rms* in R version 3.2.5. The final model was selected via a backward step-down selection process with the Akaike information criterion [40]. Performance of the nomogram was determined with the concordance index (c-index), which was estimated by analyzing the area under ROC curve and by analyzing the calibration curve comparing the nomogram-predicted survival probability versus the Kaplan–Meier estimated survival probability. Bootstraps with 1000 resamples were used for the development of the nomogram and calibration curve.

## Results

### Patient characteristics

Of the 590 included patients who were newly diagnosed with intermediate to advanced HCC and treated with TACE with or without H101 as initial therapy, 352 received TACE, and 238 received TACE with H101. The thresholds for the clinical or radiologic variables were used as the cutoff values. With the cutoff value of 3, NLR was associated with the optimal Youden index for OS prediction. After propensity score matching, 238 patients who received TACE and 238 patients who received TACE with H101 were sorted into the TACE group and the TACE with H101 group, respectively.

Baseline characteristics of patients are shown in Table 1. In the TACE with H101 group, median patient age was 54 years (range 24–85 years); 90.8% of these patients were men. In the TACE group, median patient age was 55 years (range 19–94 years); 89.9% of these patients were men. Among the selected 476 patients, 94.3% were HBsAg-positive. In the entire study cohort, large tumors with diameters equal to or larger than 5 cm and multiple tumors were most commonly seen. In both groups, the proportions of patients with large tumors and those with multiple tumors were higher than 70.0% and 60.0%, respectively. In the TACE with H101 group, 5.5% of patients had metastasis, and 30.3% had vascular invasion, whereas in the TACE group, 4.6% of patients had metastasis, and 28.6% had vascular invasion. Only IBIL and HBsAg were significantly different between two groups.

**Table 1:** Clinical and radiologic characteristics of patients with intermediate to advanced hepatocellular carcinoma (HCC) who received transarterial chemoembolization (TACE) with or without H101

Variable	Overall [cases (%)]	TACE group [cases (%)]	TACE with H101 group [cases (%)]	*P* value
Age (years)				0.420
≤60	337 (70.8)	164 (68.9)	173 (72.7)	
>60	139 (29.2)	74 (31.1)	65 (27.3)	
Sex				0.877
Man	430 (90.3)	214 (89.9)	216 (90.8)	
Woman	46 (9.7)	24 (10.1)	22 (9.2)	
WBC (×10^9^/L)				0.693
<10	449 (94.3)	223 (93.7)	226 (95.0)	
≥10	27 (5.7)	15 (6.7)	12 (5.0)	
PLT (×10^9^/L)				0.123
<100	58 (12.2)	35 (14.7)	23 (9.7)	
≥100	418 (87.8)	203 (85.3)	215 (90.3)	
ALT (U/L)				0.682
<40	173 (36.3)	79 (33.2)	94 (39.5)	
≥40	303 (63.7)	159 (66.8)	144 (60.5)	
AST (U/L)				0.924
<45	168 (35.3)	83 (34.9)	85 (35.7)	
≥45	308 (64.7)	155 (65.1)	153 (64.3)	
ALB (g/L)				0.417
<35	63 (13.2)	35 (14.7)	28 (11.8)	
≥35	413 (86.8)	203 (85.3)	210 (88.2)	
TBIL (mmol/L)				0.066
<20	381 (80.0)	182 (76.5)	199 (83.6)	
≥20	95 (20.0)	56 (23.5)	39 (16.4)	
IBIL (mmol/L)				0.011
<15	431 (90.5)	207 (87.0)	224 (94.1)	
≥15	45 (9.5)	31 (13)	14 (5.9)	
ALP (U/L)				0.773
<100	166 (34.9)	81 (34.0)	85 (35.7)	
≥100	310 (65.1)	157 (66.0)	153 (64.2)	
CRP (mg/L)				0.714
<8.2	249 (52.3)	127 (53.4)	122 (51.2)	
≥8.2	227 (47.7)	111 (46.6)	116 (48.8)	
AFP (ng/mL)				0.923
<25	164 (34.5)	81 (34.0)	83 (34.8)	
≥25	312 (65.5)	157 (66.0)	155 (65.2)	
HBsAg				0.046
Negative	27 (5.7)	19 (8.0)	8 (3.4)	
Positive	449 (94.3)	219 (92.0)	230 (96.6)	
HBV-DNA (IU/mL)				0.589
<100	112 (23.5)	59 (24.8)	53 (22.2)	
≥100	364 (76.5)	179 (75.2)	185 (77.8)	
NLR				0.916
<3	354 (74.4)	178 (74.8)	176 (73.9)	
≥3	122 (25.6)	60 (25.2)	62 (26.1)	
Tumor size (cm)				1.000
<5	130 (27.3)	65 (27.3)	65 (27.3)	
≥5	346 (72.7)	173 (72.7)	173 (72.7)	
Tumor number				0.391
Single	172 (36.1)	91 (38.2)	81 (34.0)	
Multiple	304 (63.9)	147 (61.8)	157 (66.0)	
Splenomegaly				0.177
No	311 (65.3)	148 (62.2)	163 (68.5)	
Yes	165 (34.7)	90 (37.8)	75 (31.5)	
Metastasis				0.835
No	452 (95.0)	227 (95.4)	225 (94.5)	
Yes	24 (5.0)	11 (4.6)	13 (5.5)	
Vascular invasion				0.763
No	336 (70.6)	170 (71.4)	166 (69.7)	
Yes	140 (29.4)	68 (28.6)	72 (30.3)	
Antiviral therapy				0.066
No	251 (52.7)	115 (48.3)	136 (57.1)	
Yes	225 (47.3)	123 (51.7)	102 (42.9)	

*WBC* white blood cell count, *PLT* platelet, *ALT* alanine transaminase, *AST* aspartate aminotransferase, *ALB* albumin, *TBIL* total bilirubin, *IBIL* indirect bilirubin, *ALP* alkaline phosphatase, *CRP* C-reactive protein, *AFP* alpha-fetoprotein, *HBsAg* surface antigen of hepatitis B virus, *HBV* hepatitis B virus, *NLR* neutrophil-to-lymphocyte ratio

### Overall survival of two groups

**Fig. 1:**
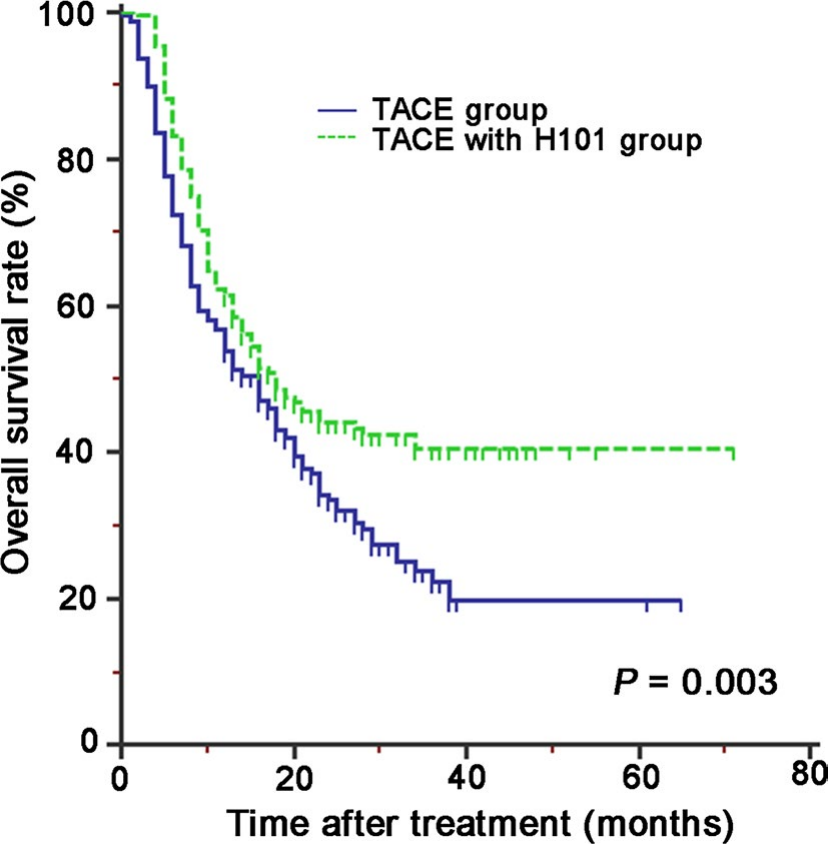
Overall survival (OS) curves estimated by the Kaplan–Meier method for patients with intermediate to advanced hepatocellular carcinoma (HCC) who were treated with transarterial chemoembolization (TACE) with or without H101

Overall survival rates of patients in the TACE group were significantly lower than those of patients in the TACE with H101 group (Fig. 1). Estimated 1-, 2-, and 3-year OS rates were 53.8%, 33.4%, and 22.4% in the TACE group and 61.3%, 44.2%, and 40.5% in the TACE with H101 group, respectively. Median OS was 14 months (range 0–65 months) for the TACE group and 17 months (range 2–71 months) for the TACE with H101 group (*P* = 0.003). These results suggest that patients who have intermediate to advanced HCC may benefit more from being treated with TACE with H101 than those patients treated only with TACE.

### Construction of the nomogram

**Fig. 2:**
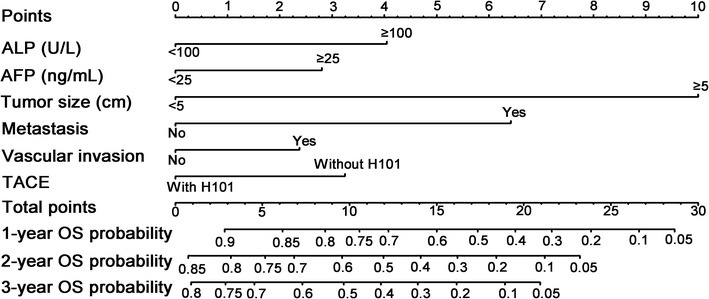
Nomogram-predicted probabilities of the 1-, 2-, and 3-year OS of patients with intermediate to advanced HCC who received TACE with or without H101. The nomogram is used by adding the points identified on the “Point” scale for six variables. The sum is located on the “Total points” scale, and a line is drawn downward to the survival axes to determine the probability of 1-, 2-, and 3-year OS. *ALP* alkaline phosphatase, *AFP* alpha-fetoprotein

**Fig. 3:**
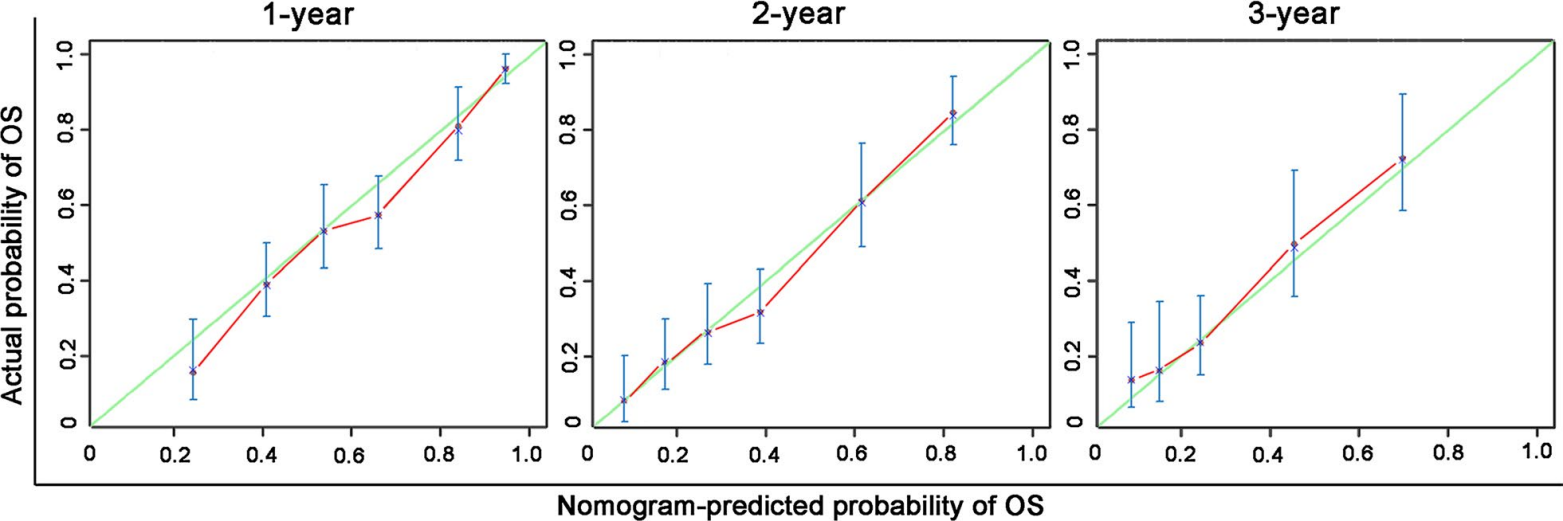
Calibration plots of the nomogram for survival prediction in the study cohort. *X*-axis represents the nomogram-predicted probability of survival; *Y*-axis represents the actual OS probability estimated using the Kaplan–Meier method. A perfectly accurate nomogram prediction model would result in a plot that the observed and predicted probabilities for given groups fall along the 45-degree line. *Dots* with *bars* represent nomogram-predicted probabilities along with 95% confidence intervals. These calibration curves showed good agreement between prediction and observation in the probabilities of 1-, 2-, and 3-year OS

The clinical and radiologic variables were included in univariate and multivariate analyses to identify the prognostic factors for OS of patients with intermediate to advanced HCC who were treated with TACE or TACE and H101. In univariate analysis, levels of AST, ALB, ALP, CRP, AFP, and NLR, tumor size, tumor number, metastasis, vascular invasion, antiviral therapy, and TACE with H101 were significantly associated with OS. These 12 risk factors were entered into the multivariate Cox regression analysis. After a stepwise removal of variables, the following six risk factors remained as significant predictors for OS: AFP, ALP, tumor size, metastasis, vascular invasion, and TACE with H101 (Table 2). Beta coefficients (BETAs) of these factors were generated using a multiple Cox regression model. Ratios of BETAs were used to determine the proportional prognostic effects of these variables in the nomogram (Fig. 2). The nomogram demonstrated good accuracy for OS prediction, with a c-index of 0.716 (95% CI 0.686–0.746). Calibration plots for the probabilities of 1-, 2-, and 3-year OS showed fair agreement between the nomogram-predicted survival and the observed survival (Fig. 3).

**Table 2:** Univariate and multivariate analyses for factors associated with overall survival of patients with intermediate to advanced HCC who received TACE with or without H101

Variable	Univariate analysis		Multivariate analysis	
	HR (95% CI)	*P* value	HR (95% CI)	*P* value
PLT (vs. <100 × 10^9^/L)	1.467 (0.993–2.166)	0.054		NI
ALT (vs. <40 U/L)	1.267 (0.993–1.617)	0.057		NI
AST (vs. <45 U/L)	2.037 (1.568–2.646)	<0.001	–	0.228
ALB (vs. <35 g/L)	0.704 (0.512–0.968)	0.031	–	0.424
TBIL (vs. <20 mmol/L)	1.213 (0.915–1.610)	0.180		NI
IBIL (vs. <15 mmol/L)	1.195 (0.809–1.765)	0.372		NI
ALP (vs. <100 U/L)	2.432 (1.859–3.180)	<0.001	1.494 (1.116–2.001)	0.007
CRP (vs. <8.2 mg/L)	2.180 (1.722–2.759)	<0.001	–	0.102
AFP (vs. <25 ng/mL)	1.547 (1.200–1.994)	0.001	1.389 (1.064–1.814)	0.016
HBsAg (vs. negative)	0.658 (0.413–1.048)	0.078		NI
HBV-DNA (vs. <100 IU/mL)	1.104 (0.876–1.392)	0.403		NI
NLR (vs. <3)	1.477 (1.145–1.904)	0.003	–	0.08
Tumor size (vs. <5 cm)	4.523 (3.156–6.481)	<0.001	3.120 (2.115–4.602)	<0.001
Tumor number (vs. single)	1.303 (1.019–1.668)	0.035	–	0.409
Splenomegaly (vs. no)	1.179 (0.928–1.499)	0.178		NI
Metastasis (vs. no)	3.668 (2.377–5.662)	<0.001	2.111 (1.342–3.321)	0.001
Vascular invasion (vs. no)	1.835 (1.443–2.335)	<0.001	1.292 (1.001–1.666)	0.049
Antiviral therapy (vs. no)	0.752 (0.596–0.950)	0.017	–	0.118
TACE (vs. without H101)	0.711 (0.563–0.896)	0.004	0.623 (0.492–0.789)	<0.001

*HR* hazard ratio, *CI* confidence interval, *NI* not included. Other abbreviations as in Table 1

– Data not shown due to no significance

## Discussion

In the present study, we compared two regimens of TACE: a conventional regimen with commonly used protocols and a regimen combining TACE with H101. We found that, compared with TACE alone, TACE with H101 extended the OS of patients with intermediate to advanced HCC. Results from multivariate Cox regression analyses showed that TACE with or without H101 was an independent prognostic factor for these patients. We developed a nomogram as an easy-to-apply model to predict the individual survival risk of patients with intermediate to advanced HCC who received TACE with or without H101. For patients who were eligible for TACE treatment, the nomogram internally showed that the combination of TACE with H101 was superior to TACE alone in terms of extending OS. For patients who previously received TACE with or without H101 as their primary treatment, the nomogram can serve as a quantitative scoring system to estimate OS.

The following mechanisms may explain the results of extended OS in patients who were treated with TACE with H101. In Asia generally, over 80% of patients with HCC are HBV-positive [41, 42]; in the present study, 94.3% of enrolled patients were positive for HBsAg. The overexpressed HBV X protein (HBx), which is produced by HBV, can inhibit *TP53* gene expression [43]. Therefore, patients with HBV-related intermediate to advanced HCC can also benefit from H101. In addition, carboplatin, which is commonly used in our institution in the TACE regimen, is similar to lobaplatin and inhibits the proliferation of human HCC cells primarily by inducing apoptosis and cell cycle arrest through the P53 apoptosis axis [44]. Furthermore, more tumor cell necrosis can be elicited by the intra-arterial injection of H101 combined with TACE, because tumor cells grow faster in the capsular end of the tumor, which is a vascular-rich area, than in a vascular-poor area such as the tumor center [45].

Our nomogram was based on routine clinical characteristics. After treating the continuous variables, including levels of AFP, ALB, and ALP, and tumor size, as dichotomous variables, significant independent risk factors were integrated to develop the nomogram for predicting the OS of the study cohort. Of all variables included in the nomogram, AFP level before TACE, tumor size, metastasis, and vascular invasion were previously recognized as prognostic factors of patients with intermediate to advanced HCC [46, 47]. The strong prognostic power of tumor size may suggest that tumor size is an irreplaceable component for worldwide guidelines of HCC [9, 36, 46, 47]. In the present study, tumor size had the highest BETA and was treated as the denominator for ratios of BETAs. Based on the ratios of BETAs, nomogram points were assigned to each parameter. Compared with published nomograms for patients with HCC who received TACE treatment [48, 49], our study included not only the conventional clinical and radiologic factors included in previous nomograms but also two additional variables: TACE with or without H101 and ALP level. The inclusion of these two additional variables may contribute to significantly greater predictive accuracy, because variables that were associated with treatment and underlying liver diseases were included in our nomogram. Our nomogram demonstrated good accuracy for OS prediction. The c-index of our study cohort was 0.716. This indicates that there was a greater than 70% probability that a patient with a high nomogram score would die earlier than a patient with a low nomogram score. Calibration plots also showed the accuracy of the OS probability that was predicted by the nomogram model. For the study cohort, calibration plot lines fell along the ideal 45-degree reference, indicating that the nomogram-predicted 1-, 2-, and 3-year survival probabilities were similar to those estimated using the Kaplan–Meier method. Combining the results of the c-index and the calibration plots, this nomogram may be considered a practical tool for risk prediction.

Our study had several limitations. First, this retrospective analysis of patients with intermediate to advanced HCC who were treated with TACE with or without H101 relied on a single-institutional dataset. Geographic and institutional heterogeneity of patients may affect these results. Second, we did not conduct an external validation for predictive accuracy of the nomogram for OS. Third, because of a high proportion of HBsAg-positive patients (94.3% of patients in the present study), this nomogram was suitable for predicting the OS of HBV-positive patients only. Finally, the combination of H101 and TACE is not widely administered in other clinical centers around the world, which limits the use of the present nomogram.

## Conclusions

In the present study, we found that patients with intermediate to advanced HCC who were treated with TACE with H101 had longer OS than those treated with TACE alone. We developed a nomogram that could predict the probability of OS for patients with intermediate to advanced HCC who were treated with or without H101. Large prospective studies are needed to confirm the survival benefit of TACE with H101 and further validate the accuracy of this prognostic nomogram.
